# The Efficiency of a Visual Skills Training Program on Visual Search Performance

**DOI:** 10.1515/hukin-2015-0051

**Published:** 2015-07-10

**Authors:** Justyna Krzepota, Teresa Zwierko, Lidia Puchalska-Niedbał, Mikołaj Markiewicz, Beata Florkiewicz, Wojciech Lubiński

**Affiliations:** 1 Department of Physical Culture and Health Promotion, University of Szczecin, Szczecin, Poland.; 2 Department of Ophthalmology, Pomeranian Medical University, Szczecin, Poland.; 3 Institute of Vision Training and Therapy, Warszawa, Poland

**Keywords:** sports vision, training, eye movements

## Abstract

In this study, we conducted an experiment in which we analyzed the possibilities to develop visual skills by specifically targeted training of visual search. The aim of our study was to investigate whether, for how long and to what extent a training program for visual functions could improve visual search. The study involved 24 healthy students from the Szczecin University who were divided into two groups: experimental (12) and control (12). In addition to regular sports and recreational activities of the curriculum, the subjects of the experimental group also participated in 8-week long training with visual functions, 3 times a week for 45 min. The Signal Test of the Vienna Test System was performed four times: before entering the study, after first 4 weeks of the experiment, immediately after its completion and 4 weeks after the study terminated. The results of this experiment proved that an 8-week long perceptual training program significantly differentiated the plot of visual detecting time. For the visual detecting time changes, the first factor, Group, was significant as a main effect (F(1,22)=6.49, p<0.05) as well as the second factor, Training (F(3,66)=5.06, p<0.01). The interaction between the two factors (Group vs. Training) of perceptual training was F(3,66)=6.82 (p<0.001). Similarly, for the number of correct reactions, there was a main effect of a Group factor (F(1,22)=23.40, p<0.001), a main effect of a Training factor (F(3,66)=11.60, p<0.001) and a significant interaction between factors (Group vs. Training) (F(3,66)=10.33, p<0.001). Our study suggests that 8-week training of visual functions can improve visual search performance.

## Introduction

One of the essential components of the perceptual mechanism in visual information processing is the ability to effectively search in order to locate a specific object among many others. This process of visual search is based on oculomotor functions, e.g. pursuit eye movements, saccadic eye movements, and steadiness of fixation. The ability to initiate a pursuit eye movement to maintain fixation of a moving object, as well as the ability to initiate an accurate saccadic eye movement for direct fixation from one object to another, are essential aspects in many daily activities (Land and Hayhoe, 2001). With regard to motor behavior, it has been indicated that the visual search ability is a factor determining the effectiveness of professional work undertaken by specialists from diverse backgrounds, e.g. drivers (Chapman et al., 2002) or beach lifeguards (Page et al., 2011).

When considering athletic performance, the efficiency of a visual search strategy is particularly important, both in individual and team sports, and among athletes of different disciplines, for example in squash (Abernethy, 1990), karate (Williams and Elliott, 1999), tennis (Singer et al., 1996; Paul et al., 2011), volleyball (Piras et al., 2014), hockey (Schwab and Memmert, 2012), soccer (Williams and Davids, 1998), etc. In many dynamic reactive sports, the speed of detection and discrimination of visual stimuli is a crucial factor in executing successful motor responses. Often, over a limited period of time, players must process and integrate complex visual information, such as the flight of a ball or movements of an opponent or a partner. The quality of the pursuit and saccadic eye movements in athletes is an essential aspect of many sport tasks. Recent reports have established that the visual motion processing of experts is much more effective than that of novices. For example, experimental findings have shown that experts have a significantly shorter response time to stimuli appearing in the peripheral field of vision (Zwierko, 2008), are more accurate in object localization and have shorter motor responses during observation in specific sport situations (Helsen and Starkes, 1999; Singer et al., 1996; Piras et al., 2010; Piras et al., 2014). Furthermore, an interesting experiment conducted by Castel et al. (2005) indicated that non-video game players did not show as fast a response time for easy and difficult visual search tasks as regular video game players. There is also some evidence indicating that playing action video games enables an improvement in many visual skills and that non-players trained on action video games significantly improved in comparison to their skills level before training (Green and Bavelier, 2003). These results indicate that participating in reactive fast-paced visual activities can have a great effect on visual search effectiveness, and suggest that such a practice could be significant in eye movement adaptation (Castel et al., 2005; Green and Bavelier, 2003). It seems that specific training of oculomotor functions could improve the visual search ability. However, experimental data have shown varied effects of exercise on visual functions.

The current study was conducted to address this issue by a systematic investigation of the effects of the visual search performance training program. On one hand, it had been indicated that the accuracy of visual search could be improved by visual search training; depending on the search strategy adopted, search performances may be altered for the better - using a systematic strategy, or for the worse - using a random strategy (Wang et al., 1997), and visual search training had been shown to be effective in the treatment of patients with homonymous hemianopia (Pambakian et al., 2004). On the other hand, some authors had indicated a lower efficiency in using visual skills training programs (Abernethy and Wood, 2001). Despite several studies focused on introducing exercises and training for visual functions (Erickson, 2007), the training program methodology used to design and improve the efficiency of visual search is one of the least analyzed topics in literature. Thus, it seemed reasonable to conduct research in this area.

Taking into account the results of several authors (Quevedo et al., 1999; Quevedo and Solé 1995; Paul et al., 2011; Balasaheb et al., 2008; Schwab and Memmert 2012; Rezaee et al., 2012) indicating the effectiveness of training of visual functions, we conducted an experiment in which we expected confirmation of the efficacy of specifically targeted training in the development of the visual search ability. We hypothesized that the efficiency of visual functions used in visual search was higher in people following a training program in order to improve these functions than in individuals not involved in any such training.

Hence, we tried to analyze whether, how long and to what extent a training program of visual functions would improve the visual search ability.

## Material and Methods

### Participants

The study involved 24 healthy physically active female students of physical education from the Szczecin University. The subjects were divided into two groups. The experimental group consisted of 12 women (mean age 21.5 ± 1.4 years) while the control group included the remaining 12 women (mean age 20.5 ± 1.8 years). All participants showed a similar level of physical fitness and did not participate in any activities which might affect the results of this study. Prior to the experiment, the subjects were tested in the Department of Ophthalmology in the Pomeranian Medical University in Szczecin, in order to exclude those with any vision faults which would make participation in the experiment impossible. The tests included: distance visual acuity, refractive errors, anterior segment and fundus of the eye examination, eye position, study of the eye motility into each of the six cardinal positions of gaze, accommodation and heterophoria related reseach.

The subjects voluntarily participated in this research, signed a written informed consent form and were able to withdraw from the study at any time. The local Bioethical Committee in Szczecin (No° 11/KB/V/2013) approved the research project.

### Measures

In addition to the regular sports and recreational activities of the physical education curriculum, the subjects from the experimental group also followed an 8-week training program of visual functions 3 times a week for 45 minutes, and underwent a Signal Test using the Vienna Test System (Schuhfried, Austria) four times: before the experiment, after 4 weeks of the experiment, on completion of the experiment, and 4 weeks after its cessation. In contrast, subjects from the control group participated in the same sports and recreation activities of the physical education curriculum as the experimental group, undertook the four Signal Tests, but were not included in any additional visual function training program.

The Signal Test measured the visuospatial differentiation of a relevant signal within irrelevant signals. This test evaluates the visual detailed registration of complex stimuli under time pressure over a longer period of time by constantly engaging oculomotor functions, e.g. pursuit and saccadic eye movements and steadiness of binocular fixation during the task. In our experiment, we utilized a standard version S1 with white signals (dots) on a black background. Dots were displayed over the whole screen area, pseudo-randomly some of the dots vanished and other came into view. The participants were asked to perform a key-press response to a programmed stimulus constellations whenever it took place. This critical stimulus constellation was created by four dots which together formed a square (Picture 1). Each of the subjects was prepared for the main task by participating in pre-tests that enabled them to familiarise themselves with the apparatus and the task itself. The total testing time was about 20 minutes (including instruction and practice phases). The main variables calculated were the numbers of correct, omitted and incorrect reactions, and the median reaction time as a measure of the speed of the detection process.

### Procedures

The subjects from the experimental group followed an 8-week training program for visual functions, 3 times a week for 45 minutes (Erickson, 2007). At the beginning and at the end of each training session participants performed some relaxation exercises. During the workout they practised visual search for 20 minutes.

Based on literature in this field (Erickson, 2007), we created a training program with visual search exercises designed specifically for the experiment. The main objective of the proposed program was the use of simple and inexpensive exercises inducing visual function improvement. The important factor was that training and exercises could be conducted by subjects in their own homes or while training in different places, without any special equipment.

The training exercises used visual search arrays where eye movements in six directions of fixation were practiced, fixation variability was used and exercises concerning searching of moving objects were performed. Exercises were carried out within a time limit, both in static and dynamic positions. Examples of exercises used in training of visual search functions are presented below. Each exercise was applied for 1 min, followed by a rest period. The training program was controlled by a psychologist and a vision improvement teacher.

The following visual training procedure was applied:

– Alternate reading of letters placed in two columns (left and right side of the page) as quick as possible, one from the right and one from the left column, from the top to the bottom, or vice versa.
– Searching for a specific letter from the right, then the left column (with the quickest simultaneous pointing with a finger to the letter).
– The participant calls out (from charts with random letters/numbers) the first and the last letter/number of each row until they reach the bottom of the charts. After successful completion of this task, the person is requested to call out the second letter/number from both the beginning and end of each row, and then the third letter/number from both the beginning and end of each row, etc., or some other challenging saccade patterns.
– Following reading of letters from each column (in the vertical and horizontal position) of a card, a partner holding the card moves one meter further away. The partner then moves the card in both vertical and horizontal directions, drawing circles in the air.
– Finding letters as quickly as possible, creating a specified word from a paper on which the letters are randomly scattered.
– Searching out numbers from a whole square divided into four quadrants with randomly positioned digits: in order, i.e. 1, 2, 3 etc., or 2, 4, 6, 8 etc., or in other configurations specified by a partner in each quadrant or in the whole square.
– Moving a finger toward the arrow being named from charts with arrows pointing in random directions (right, forward, and so on).
– Finding and pointing with a finger toward a pattern, among other models outlined on a card named by a partner.
– Finding details distinguishing two drawings.
– Following a moving object held by a partner, using a dice while reading the number of spots on the dice (or a ball with a letter or number written on it).
– Following a partner’s finger plotting different patterns in the air (number eight, the sign of infinity, circle, spiral, etc.).
– Searching for dice with a specified number of spots, among dice scattered in a 1 m diameter circle.

### Statistics

The dependent measures (visual detection time and number of correct reactions) were analyzed separately using a two-way repeated measure analysis of variance (ANOVA) to test the significance of between-subject factors (Group – experimental and control) and within-subject factors (Training: pre-training - Sig_0; after 1 month of training - Sig_1; after 2 months of training - Sig_2; control conditions - Sig_c; 1 month after the experiment). In order to determine the significance of intra- and intergroup differences, ANOVA contrast analysis was used.

## Results

### Effects on visual detecting time

**Figure 1:**
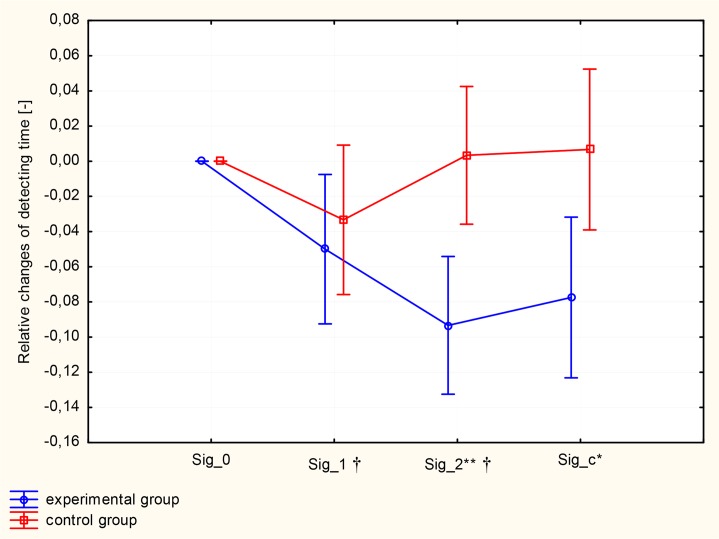
Relative changes of visual detecting time in experimental and control groups. Pre- and post-training values are presented as means and ±SEM. A significant difference (p<0.01) between the experimental group and control group after 2 months of perceptual training (Sig_2) is denoted with ^**^ and at control conditions (Sig_c, p<0.05) is denoted with ^*^. Significant intragroup differences (p<0.05) in the experimental group (Sig_0 vs. Sig_1, and Sig_1 vs. Sig_2) are denoted with (†).

In our experiment, we analyzed the influence of two factors on visual detection time: (1) Group (experimental vs. control group) and (2) Training. For the visual detecting time changes, the first factor, Group, was significant as a main effect (F_(1,22)_=6.49, p<0.05), and the second factor, Training, was also significant as a main effect (F_(3,66)_=5.06, p<0.01). The interaction between two factors (Group vs. Training) of perceptual training was F_(3,66)_=6.82 (p<0.001). Perceptual training significantly differentiated the plot of changes in visual detection time when the experimental and control groups were compared. ANOVA contrast analysis indicated a differential effect between the experimental and control groups after two months of perceptual training (Sig_2: F_(1.22)_=13.10, p<0.01) and 4 weeks following the research accomplishment (Sig_c: F_(1.22)_=7.29, p<0.05). ANOVA contrast analysis also indicated significant intra-group differences among the experimental subjects after one month of perceptual training ((Sig_0 vs Sig_1: (F_(1.22)_=5.97, p<0.05)) and (Sig_1 vs Sig_2: (F_(1.22)_=6.38, p<0.05)). The visual detection time improved during and after training stimulation in comparison to the baseline (Sig_0 vs. Sig_2: F_(1.22)_=24.44, p<0.001). The plot of interactions for the analyzed factors, with regard to visual detection time, is illustrated in Figure 1.

### Effects on the number of correct reactions

**Figure 2:**
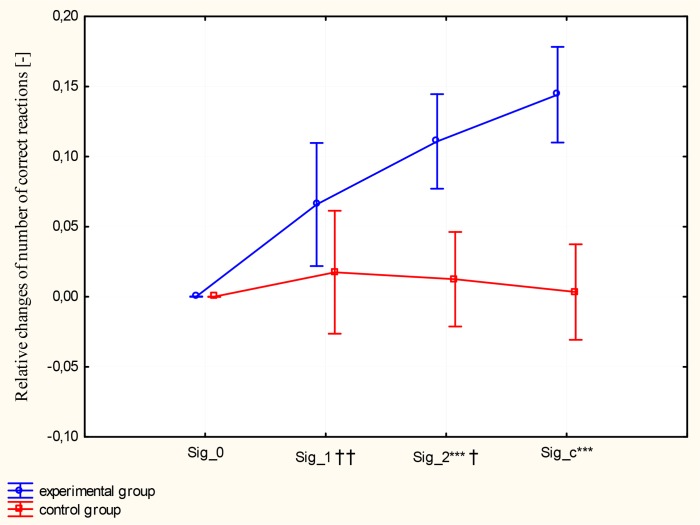
Relative changes of the number of correct reactions in experimental and control groups. Pre- and post-training values are presented as means and ±SEM. A significant difference between the experimental and control group after 2 months of training (Sig_2, p<0.01) is denoted with ^**^ and at control conditions (Sig_c, p<0.001) is denoted with ^***^. Significant intragroup differences (p<0.01) in the experimental group (Sig_0 vs. Sig_2) are denoted with (††), and between Sig_1 vs. Sig_2 (p<0.05) are denoted with (†).

There was a main effect of a Group factor (F_(1,22)_=23.40, p<0.001), a main effect of a Training factor (F_(3,66)_=11.60, p<0.001), as well as a significant interaction between the two factors (Group vs. Training) (F_(3,66)_=10.33, p<0.001) in the number of correct reactions. ANOVA contrast analysis showed inter-group (experimental vs. control group) differences after 2 months of training (Sig_2, F_(1.22)_=18.31, p<0.001) and 4 weeks following the experiment (Sig_c F_(1.22)_=36.69, p<0.001) (Figure 2). ANOVA contrast analysis indicated intra-group differences in the experimental group ((Sig_0 vs. Sig_1: F_(1.22)_=9.64, p<0.01) and (Sig_1 vs. Sig_2 F_(1.22)_=5.22, p<0.05)). The number of correct reactions improved during and after training stimulation in comparison to the baseline (Sig_0 vs. Sig_2: F_(1.22)_=46.44, p<0.001).

## Discussion

The purpose of the present study was to analyze whether, for how long and to what extent a training program for visual function would improve the visual search ability.

We expected that exercise methodology used in our visual training program could be useful to implement into athletic training. The results of the present study showed that 8 weeks of intensive perceptual training significantly improved visual search variables. Additionally, the results of the retention test confirmed the visual training effects. The present study showed a new point of view on methodology of visual training which consisted of orthoptic, sport and psychological aspects. Our experiment confirmed that visual search ability is trainable. In our opinion, the implementation of a visual training program is important for athletes in reactive, fast-paced sports.

The results from the present study are in line with previous research demonstrating beneficial effects of visual functions training in athletes, after a 6-week period (Paul et al., 2011; Balasaheb et al., 2008; Schwab and Memmert, 2012), after 8 weeks (Rezaee et al., 2012) or even after three months of training (Quevedo and Solé, 1995). Some authors did not confirm an improvement in visual skills, since no increase in vision performance between the groups (pre- and post training) after the given period of four weeks of the visual training program was found in their research (e.g. Abernethy and Wood, 2001). Those authors suggested that perhaps 4 weeks of practice may have not been sufficient to result in statistically significant training effects. The results of our research confirm that 4 weeks of training induce significant changes in the experimental group when compared to the control one. Other researchers (Schuster et al., 2013) reported that when visual search involved detecting heterogeneous or otherwise unpredictable stimuli, perceptual training may improve the speed and accuracy of visual search. Nevertheless, it is worth mentioning that there is no ambiguity in the scientific world connected with the effectiveness of training of visual functions. Quevedo et al. (1999) draw attention to the fact that the effectiveness of visual function training may depend on the duration, frequency and even the number of people participating in the exercises. Moreover, it is important to note that the tightly controlled visual searches performed in the laboratory can lead to significantly different results when compared to field searches (Clark et al., 2012). Hence, it is possible that discrepancies in the obtained results may be due to a different period of training and various research methods applied, as well as due to differences in the level of physical fitness among the participants. For instance, research carried out by Rezaee et al. (2012) concerning an 8-week training program showed a significant effect on eye saccadic movement measured by a swinging ball exercise, in groups with visual training only and in groups with additional sport specific training (for instance, table tennis and basketball). However, Rezaee et al. (2012) also point to the fact that the specificity of the sport can be a decisive factor in improving visual skills, as they observed an improvement in visual skills (e.g. saccadic movement) in the group where subjects practiced exclusively table tennis, in contrast to basketball players in whom such an improvement was not observed.

In turn, Balasaheb et al. (2008) clearly indicated superior eye movement skills only in the experimental group, who had followed six weeks of visual training. They observed most significant improvements in horizontal and vertical saccadic eye movements and reaction in the experimental group after 6 weeks of a visual training program which consisted of exercises performed 3 days a week. They did not find such results in the two control groups: 1) the placebo group of which subjects were given simple reading materials and watched television cricket matches for 6 weeks, and 2) the control group who followed routine cricket training only. However, slightly different results were obtained by Paul et al. (2011) in tennis players divided into three groups: an experimental, placebo and control group. During 8 weeks of vision training (3 days a week for 30 min each) the authors noticed a significant improvement (p<0.001) in pre- and post-training reaction time in the experimental group, in contrast to the results of both the placebo group (of which subjects were requested to read materials and watch television tennis matches) and the control group. However, as far as the horizontal and vertical saccadic eye movements are concerned, in all the groups a significant improvement (p<0.05) was observed, though the most significant improvement was exhibited by the experimental group (p<0.001). The results suggest that participating in reactive, fast-paced sports training has a positive effect on visual functions. Similar conclusions were drawn by Zwierko et al. (2014).

Another issue still not fully recognized is the time in which a decrease in efficiency occurs after completing training. In our research we observed that one month after the completion of visual function training, the number of correct reactions in experimental groups remained at the same level and did not significantly change (increase or decrease). Detection time, despite a slight deterioration in performance, was still higher than before the experiment after 4 weeks of the research. Therefore, it should be considered that the applied training program allowed a relatively stable increase in the efficiency of tested functions in the experimental group. It is worth mentioning that some authors (Passamonti et al., 2009) stated that audio-visual training stimulating the superior colliculus could develop a better organized visual exploration pattern in patients with hemianopia due to the use of efficient oculomotor strategies. Furthermore, the improvement still remained at the same level one year afterwards, during a follow-up control session, thus showing long-term treatment effects regarding the oculomotor system.

The latest research concerning eye exercises using a modified rapid serial visual presentation (Di Noto et al., 2013) points out the usefulness of eye exercises to improve cognitive performance in tasks connected with attention and memory during a short training course (one month). The authors indicate that the improvement in performance could be explained by the fact that the common cortical network mediates attention, cognition and oculomotor movement, and undergoes very short-term neuronal plasticity. It has also been documented that it is possible to learn in an attentive tracking task, despite the fact that the visual stimuli are complex and the task requires a lot of attention (Makovski et al., 2008). Furthermore, the findings of some authors suggest that saccades can effectively improve the attentional control (Edlin and Lyle, 2013). Hence, we suppose that our results can probably also be explained by these processes.

In addition, Reeves and Swenson (1981) stated that when moving the eyes some muscles increase and others decrease their activities. It was observed (Glimcher, 2002) that a mixture of static and dynamic forces create eye movements. In order to keep the eye at a given position, a static force has to be produced by all the muscles involved. An adequate level of strength is needed to counteract the work of all the other muscles. Moving the eyes from one eccentric position to the other requires a dynamic force which overcomes resistance of the muscles. This enables the eye to produce a movement. While a static force must exist when the eye is stationary, a dynamic force must only be used during a real movement of the eye. However, Reeves and Swenson (1981) state that eye movements below or above the horizontal plane are complicated, requiring at least the activation of pairs of muscles, while horizontal (lateral and medial) eye movements are quite simple. These movements are produced by increased activation of one muscle: the lateral rectus or the medial rectus.

In conclusion, despite the fact that results of our study suggests that 8 week training of visual functions can improve visual skills, particularly visual search, the issue of duration of training of visual functions is not completely clear. It is important that in the future, researchers focus on developing procedures that would allow the use of an optimal volume of training exercises supporting visual effects. Some results of previous research (Pambakian et al., 2004) analyzed the transfer of visual search training into activities of daily living and subjective improvements in patients with homonymous hemianopia, while other authors considered incorporating visual function exercises into regular sports training (Schwab and Memmert, 2012; Balasaheb et al., 2008). However, in the scientific world this problem is also still not completely recognized, and further research in this area is recommended.

Based on the aforementioned factors, we have undertaken further research which will concentrate on determining whether and to what extent the improvement of visual functions using exercises proposed in this paper affects the effectiveness of motor abilities, which may have important implication in sports training.

## Figures and Tables

**Picture 1:**
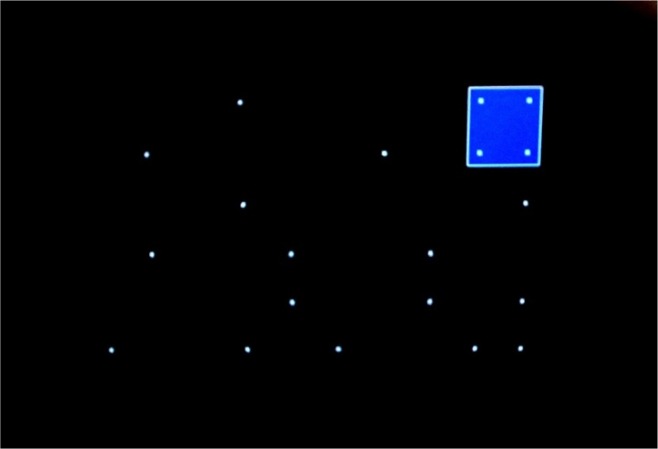
White signals (dots) on a black background and critical stimulus constellation in the Signal Test
